# Exercise therapy, manual therapy, or both, for osteoarthritis of the hip or knee: a factorial randomised controlled trial protocol

**DOI:** 10.1186/1745-6215-10-11

**Published:** 2009-02-08

**Authors:** J Haxby Abbott, M Clare Robertson, Joanne E McKenzie, G David Baxter, Jean-Claude Theis, A John Campbell, the MOA Trial team

**Affiliations:** 1 Centre for Physiotherapy Research, School of Physiotherapy, University of Otago, Dunedin, New Zealand; 2 Dept of Medical & Surgical Sciences, Dunedin School of Medicine, University of Otago, Dunedin, New Zealand; 3 Dept of Preventive and Social Medicine, Dunedin School of Medicine, University of Otago, Dunedin, New Zealand; 4 Monash Institute of Health Services Research, Monash University, Melbourne, Australia

## Abstract

**Background:**

Non-pharmacological, non-surgical interventions are recommended as the first line of treatment for osteoarthritis (OA) of the hip and knee. There is evidence that exercise therapy is effective for reducing pain and improving function in patients with knee OA, some evidence that exercise therapy is effective for hip OA, and early indications that manual therapy may be efficacious for hip and knee OA. There is little evidence as to which approach is more effective, if benefits endure, or if providing these therapies is cost-effective for the management of this disorder. The MOA Trial (Management of OsteoArthritis) aims to test the effectiveness of two physiotherapy interventions for improving disability and pain in adults with hip or knee OA in New Zealand. Specifically, our primary objectives are to investigate whether:

1. Exercise therapy versus no exercise therapy improves disability at 12 months;

2. Manual physiotherapy versus no manual therapy improves disability at 12 months;

3. Providing physiotherapy programmes in addition to usual care is more cost-effective than usual care alone in the management of osteoarthritis at 24 months.

**Methods:**

This is a 2 × 2 factorial randomised controlled trial. We plan to recruit 224 participants with hip or knee OA. Eligible participants will be randomly allocated to receive either: (a) a supervised multi-modal exercise therapy programme; (b) an individualised manual therapy programme; (c) both exercise therapy and manual therapy; or, (d) no trial physiotherapy. All participants will continue to receive usual medical care. The outcome assessors, orthopaedic surgeons, general medical practitioners, and statistician will be blind to group allocation until the statistical analysis is completed. The trial is funded by Health Research Council of New Zealand Project Grants (Project numbers 07/199, 07/200).

**Discussion:**

The MOA Trial will be the first to investigate the effectiveness and cost-effectiveness of providing physiotherapy programmes of this kind, for the management of pain and disability in adults with hip or knee OA.

**Trial registration:**

Australian New Zealand Clinical Trials Registry ref: ACTRN12608000130369.

## Background

Non-pharmacological, non-surgical interventions, such as the treatments offered by physiotherapists, are recommended as the first line of treatment for hip and knee osteoarthritis (OA) [1-3], however there is still insufficient evidence about many such interventions to make specific recommendations regarding management of this disorder [4,5]. Given the prevalence of OA, its economic and human burden, and accumulating evidence supporting the effectiveness of various physiotherapy interventions for patients with OA of the hip or knee, further research is warranted [5]. Two common forms of physiotherapy intervention are exercise therapy and manual therapy.

Although it is well established that various forms of exercise are effective in reducing pain and increasing physical function in people with knee OA, there is still little knowledge about which forms of exercise are most effective [4-8]. Little is known about whether effectiveness endures beyond the medium term, with few studies following up to 12 months, and none beyond 15 months [9]. Few trials have investigated the relative benefits of differing exercise approaches: systematic reviews of exercise therapy for the osteoarthritic knee have included randomised controlled trials, which are clinically diverse, with variability in the interventions employed [4,7,9,10]. However, one meta-analysis of trials on strengthening exercises concludes that, to get the maximum benefit of strengthening exercises, it is necessary to also include range-of-motion and stretching exercises in a multi-modal approach [11]. There are no trials that we know of investigating a supervised, individualised, multi-modal programme of exercise therapy for OA of the knee, although clinicians consider this to characterise the 'ideal standard of clinical practice' [12].

Regarding OA of the hip, there have been very few randomised controlled trials of exercise therapy, however what data there are indicate the benefits may be similar to those found for knee OA [5,11,13-15]. Although there are no systematic reviews available specific to exercise therapies for hip OA, reviews that have summarised either hip and/or knee OA identified no trials of strengthening exercises or of land-based supervised exercise for people with OA of hip and had not undergone hip joint replacement surgery [8,9,11,12,15]. Two more recent trials in the literature indicate that a programme of multimodal exercise training and lifestyle advice [14] and a multi-modal strengthening, stretching, gait training and range of motion exercise programme [16] may be effective for reducing pain and improving function in patients with hip OA, however effect sizes were small in both trials. Again, little is known about whether effectiveness endures beyond the medium term.

New developments in manual physiotherapy have demonstrated very promising improvements in pain and physical function [16-19], but effectiveness has not yet been definitively established [5]. There is one clinical trial in the literature of manual physiotherapy for hip OA: compared with exercise physiotherapy, manual physiotherapy resulted in superior improvements in pain and physical function, that endured to six months follow-up [16]. Deyle and colleagues have conducted two trials of manual physiotherapy for knee OA [17,18]. In the first, a multi-modal intervention of individually tailored manual therapy plus stretching, strengthening, range of motion and home exercises resulted in clinically significant superior outcomes compared with a placebo control group [18]. In the second, the multi-modal intervention of individually tailored manual therapy plus stretching, strengthening, range of motion and home exercises resulted in clinically significant superior outcomes compared with home exercises alone [17]. In both studies, benefits were still evident at the 12-month follow-up.

Our project is an extension of these recent studies [16-18], and is designed to answer questions that have not been addressed in the literature to date. None of the three previous controlled trials of manual physiotherapy has directly compared manual therapy interventions with usual care. We have identified no trials directly comparing an individually tailored, supervised multi-modal exercise therapy programme with usual care, or to individually tailored manual therapy. No trials have investigated if there is a synergistic, antagonistic or neutral effect when both exercise and manual therapy are employed. Few of the previous trials have assessed the effectiveness of their interventions beyond a short to medium term. The MOA Trial responds to the dearth of economic analysis studies in this field, and will report cost-utility over a 24-month timeframe.

The MOA Trial will investigate the long-term effectiveness and cost-effectiveness of both a multi-modal, individualised, supervised exercise therapy programme, and an individualised manual therapy programme, for the management of pain and disability in adults with hip or knee OA. This publication outlines the design and analysis plan for the MOA Trial.

### Specific aims

The specific aims of this trial are to establish if:

1. Exercise therapy versus no exercise therapy improves disability at 12 months in adults with hip or knee OA.

2. Manual therapy versus no manual therapy improves disability at 12 months in adults with hip or knee OA.

3. Provision of physiotherapy programmes in addition to usual care to adults with hip or knee OA is more cost-effective than usual care alone at 24 months.

In addition, we will test for differences in the secondary outcomes at 9 weeks, 6 months, and 12 months, and for an interaction between the interventions for disability at 12 months. The secondary outcomes include physical performance tests, patients' global assessment, and joint replacement surgery (Table 1).

**Table 1:** Outcome measures

Primary Outcome measure*	Data collection instrument
WOMAC composite score	WOMAC-3.1 patient-rated questionnaire [30-32]

**Secondary Outcome measures**	

Timed up-and-go	Physical test [38,39]
30 second sit-to-stand	Physical test [37]
40 m self-paced walk	Physical test [32,39]
WOMAC subscales: A) Pain; B) Stiffness C) Physical Function;	WOMAC-3.1 [33,34] patient-rated questionnaire
Numeric pain rating	NPRS [33,34] patient-rated questionnaire
Self-efficacy and pain beliefs	The Pain Belief Screening Instrument [40,41]
Depression	The two-item case-finding instrument [42]
Patient's global assessment	GROC [33,34,36] patient-rated scale
OARSI response criteria	Composite of WOMAC, NPRS, GROC [34]
American College of Rheumatology criteria for diagnosis of OA	Clinician-rated criteria [22-24,45,46] from physical tests and symptoms
New Zealand National Clinical Priority System score	Clinician-rated criteria [47];
Adverse events	MOA field team audit; ODHB records; self-report questionnaire
Overall health status^†^	SF-12 general health survey [43]
Surgical intervention^†^	Self-report questionnaire; health system records [44]
Healthcare consumption and related costs^†^	Self-report questionnaire; health system records

*The primary end-point for data analysis is 12 months. All outcome measures will be undertaken at baseline, 9 weeks, 6 months, 1 year and 2 years, with the exception of patients' global assessment and OARSI response criteria, which will not be assessed at baseline. ^†^The primary endpoint for cost-utility and surgical intervention is at 2 years. WOMAC = Western Ontario and McMaster osteoarthritis index; NPRS = numeric pain rating scale; GROC = global rating of change; OA = Osteoarthritis; OARSI = Osteoarthritis Research Society International; MOA = the Management of OsteoArthritis Trial; ODHB = Otago District Health Board.

## Trial design and methods

**Figure 1:**
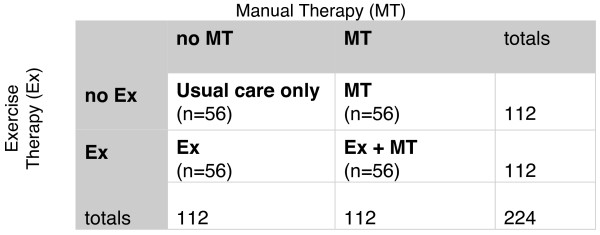
**2 × 2 factorial trial design**. Note: All groups receive usual medical care.

This project is a 2 × 2 factorial randomised controlled trial (Figure 1). We have chosen a factorial design because it is an efficient way to evaluate more than one intervention in a single trial [20,21]. This is because all participants are included in each of the primary analyses, whereas in a trial of, say, three parallel arms evaluating two different interventions compared with a control arm, only two thirds of participants are used for each comparison. However, the sample size benefits gains of this design only occur when the assumption of no interaction between the two interventions is met. We do not anticipate any interaction effects since these are uncommon [21], even in drug trials [20]. However, we will report an estimate of the interaction effect as is recommended for all factorial trials [20] (see Data Analysis, below).

### Participants

We will recruit 224 participants through two sources: a) patients attending general practitioners (GPs) with hip or knee OA; and b) patients attending the Department of Orthopaedic Surgery (Outpatient Clinic, Dunedin Hospital, New Zealand) for an orthopaedic consultation for consideration of hip or knee joint replacement surgery.

To be eligible, participants must meet clinical criteria for diagnosis of OA of the hip or knee according to American College of Rheumatology criteria [22-24].

Exclusion criteria will include:

• previous knee or hip joint replacement surgery of the affected joint;

• any other surgical procedure of the lower limbs in the previous 6 months;

• rheumatoid arthritis;

• initiation of opioid analgesia or cortico-steroid or analgesic injection intervention for hip or knee pain within the previous 30 days;

• uncontrolled hypertension or moderate to high risk for cardiac complications during exercise [25];

• physical impairments unrelated to the hip or knee preventing safe participation in exercise, manual therapy, walking or stationary cycling, such as: vision problems that affect mobility, body weight greater than 155 kg, neurogenic disorder, primary or significantly limiting back pain, advanced osteoporosis, or inability to walk 10 metres without an assistive device;

• inability to comprehend and complete study assessments or comply with study instructions;

• stated inability to attend or complete the proposed course of intervention and follow-up schedule.

**Figure 2:**
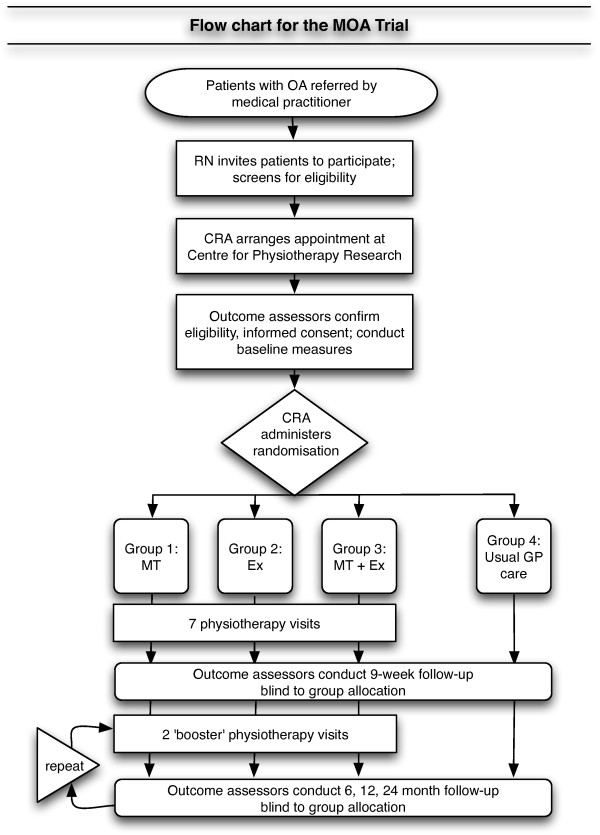
**Diagram of participant flow**. OA = osteoarthritis; RN = Research Nurse; CRA = Clinical Research Administrator; MT = manual physiotherapy; Ex = exercise physiotherapy; GP = general medical practitioner.

Figure 2 shows a diagram illustrating the expected flow of participants through recruitment, assessment and intervention. A research nurse will identify potential participants at the Dunedin Hospital Orthopaedic Clinic point of entry. The research nurse will initially screen each potential participant against the inclusion and exclusion criteria by chart review and questioning by telephone. We will keep a screening log, recording the criteria eliminating all those found to be ineligible. Willing potential participants will be given an appointment at the Centre for Physiotherapy Research, where researchers will obtain informed consent and baseline measures.

Researchers at the Centre for Physiotherapy Research will evaluate each potential participant against the inclusion and exclusion criteria by clinical history, physical examination and review of self-report questionnaires. Participants will then be randomised (refer to randomisation and allocation concealment, below).

In the event of recruitment numbers falling behind targets, we will seek ethical approval to recruit people with hip or knee OA by advertising in the community. Previous research indicates there may be no clinically or statistically significant differences in most important baseline measures or mean effects of treatment between patients referred by GPs for clinical consultation versus people recruited from the community via newspaper advertisements [26].

#### Randomisation and allocation concealment

Participants will be randomised using TENALEA, which is an online, central randomisation service, currently in deployment phase with a grant from the e-TEN programme of the European Union (LSHC-CT-510736). This Trans-European Network for Clinical Trial Service provides a secure randomisation service over the internet. The TENALEA service will generate and hold the randomisation schedule. Allocation concealment will be ensured, as the service will not release the randomisation code until the patient has been recruited into the trial, which takes place after all baseline measurements have been completed.

Randomisation will be stratified by condition (hip or knee). Within each stratum, participants will be randomised to one of the four intervention groups using block allocation. The block size will be subject to random variation.

#### Blinding

Outcome assessors will be blind to group allocation, and will not be involved in providing the interventions. The orthopaedic surgeons and GPs managing the participants' care will be blind to group allocation; physiotherapists delivering the intervention cannot. The participants will be informed they are in a "physiotherapy" or the "usual care" group, and the type of physiotherapy intervention (manual, exercise, or both) will not be specified. The statisticians conducting the statistical analyses will be blind to group allocation until the analyses are completed.

### Interventions

Protocols for the physiotherapy interventions will be described in a Manual of Standard Operating Procedures (MSOP), available from the principal investigator (JHA). Guidelines for individual tailoring of activity prescription and progression will be described within these protocols. Secondarily, additional interventions will be individually tailored as needed, according to the results of a standardised physical examination that will include tests and measures to assess impairments in physical function (e.g. muscle strength, muscle length, joint range of motion). Impairments revealed will be matched explicitly to interventions, described in the MSOP, intended to directly address each impaired physical function. In this manner, the plan of care for each patient is individually tailored from a standardised menu of interventions, using a defined algorithm, in the categories of 'exercise therapy' and 'manual therapy' (described below). Individualised home exercise instructions, plus compliance logs, will also be provided. The methodology will therefore be consistent with the 'ideal standard of clinical practice' [12] and the research designs of previous studies [16-18].

Participants randomised to receive trial physiotherapy will receive nine treatment sessions: seven in the initial nine weeks of the trial and two 'booster' sessions at week 16. Given the chronic, progressive nature of the disease, we will recommend participants return to receive additional 'booster' doses of two sessions every four months, as is recommended by current best evidence, previous investigators and expert opinion [9,17,27,28].

#### Usual care

The control group will receive no trial physiotherapy. All participants will continue to receive the usual, normal routine care offered by their own GP and other healthcare providers. In this way, this trial evaluates the effectiveness of physiotherapy care *in addition to *usual medical care.

We will send a letter to each participant's GP informing her or him that the patient is participating in the trial, but will not reveal which group the patient is in and will request that the GP not ask. We will also request that the GP contact the researchers before adding physiotherapy to the patient's plan of care during the trial. The researchers will discourage contamination of the 'usual care only' group by referral to physiotherapy within the 9-week intervention phase, however it would be unethical and unreasonable to recommend physiotherapy be withheld from the 'usual care only' group throughout the follow-up period of the trial. We will measure potential contamination with non-trial physiotherapy by participant self-report questionnaire.

All participants will continue to receive their usual care at the Dunedin Hospital Orthopaedic Clinic, by consultant orthopaedic surgeons blind to group allocation. Participation in the trial will not affect patients' prioritisation for, or access to, joint replacement surgery.

#### Exercise physiotherapy

The exercise therapy protocol will primarily consist of a multi-modal, supervised programme of warm-up/aerobic, muscle strengthening, muscle stretching, and neuromuscular control exercises. Secondarily, additional exercise therapy interventions will be prescribed individually for each participant on the basis of the physical examination findings, from a limited list of interventions defined in the MSOP. These will be informed by evidence-based best practice [5,9-13,16-18,29]. The protocol does not allow therapist-applied manual forces. Participants will be instructed in an individualised home exercise programme of warm-up/aerobic, muscle strengthening, muscle stretching, and neuromuscular control exercises as detailed in the MSOP.

#### Manual physiotherapy

The manual therapy protocol will consist primarily of procedures intended to modify the quality and range of motion of the target joint and associated soft tissue structures. Secondarily, additional manual therapy interventions will be prescribed individually for each participant randomised to this intervention on the basis of the physical examination findings, from a limited list of interventions. These will be informed by evidence-based best practice [16-19]. For our purposes, we define manual therapy as the application of therapist-applied manual forces in procedures intended to modify the quality and range of motion of the target joint and soft tissue structures. The protocol does not allow muscle strengthening or neuromuscular control exercises. Participants will be instructed in an individualised home exercise programme of joint range of motion exercises.

#### Combination therapy

This protocol will consist of a combination of both exercise therapy and manual therapy interventions, as described above.

### Outcome measures

#### Primary

The primary outcome will be disability at one-year follow-up using the WOMAC (Western Ontario and McMaster osteoarthritis index). The WOMAC is a widely used, reliable, valid and responsive measure of outcome in people with osteoarthritis of the hip or knee [30-32].

#### Secondary

We will also include the outcome measures recommended by OMERACT-OARSI guidelines [33,34]. These require measures of pain, physical function and patient global assessment. We will assess pain using the WOMAC pain sub-scale and numeric pain rating scale [35]; physical function using the WOMAC physical function sub-scale; and patient global assessment using a 15-point global rating of change [36] (Table 1). We will also assess change in physical performance tests: the timed up-and-go, 30-second sit-to-stand, and 40 metre self-paced walk tests [32,37-39] and psychological function [40-42]. We will assess overall health status using the SF-12 (Quality Metric, Inc., Lincoln, Rhode Island, USA) [43], and will measure health care consumption costs, including surgical interventions, by records audit and patient self-report questionnaire [44]. We will track and classify adverse events. We will continue to assess the American College of Rheumatology criteria for the diagnosis of OA of the hip or knee [22-24,45,46]; the New Zealand National Clinical Priority System score [47] (where available); and exercise compliance.

#### Follow-up

A summary of the follow-up schedule is shown in Table 1. Assessments will be performed by researchers at baseline, 9 weeks, 6 months, 1 year, 2 years and, if future funding permits, subsequent years.

### Sample size

The primary endpoint in this trial is disability at 12 months, measured using the numeric rating scale version of the WOMAC scale. We calculated the sample size to detect a difference of 28 points for each of the main effects, namely, exercise therapy versus no exercise therapy, and manual therapy versus no manual therapy. We regard this as the minimum clinically important difference between groups in patients with moderate OA [17,18,32,48-50]. Assuming a standard deviation of 50 points [17,18], and a type I error rate of 5%; a sample of 45 participants per intervention group (90 per row (Figure 1)) will be sufficient to detect the differences with approximately 95% power. With a sample of this size, the predicted width of the 95% confidence interval for each intervention effect will be approximately ± 14.6 points [51]. Allowing for 20% attrition, we plan to recruit 56 participants per group, providing a total of 224 participants.

Using the assumptions above, a sample of this size will have limited power of approximately 46% to detect an interaction between exercise therapy and manual physiotherapy. However, these calculations may be conservative since we have not incorporated the stratification variable, or correlation between baseline and follow-up measures, due to lack of prior information.

### Data analysis

#### Analyses subsets

The primary analysis of the data will be undertaken using the principle of intention-to-treat (ITT) [45]. Our ITT analysis will include all participants, including those who are not fully compliant and those with missing outcome data. While we plan to implement procedures to minimise loss to follow-up and patient withdrawal, we expect to observe some attrition. We plan to employ multiple imputation to handle missing data in the analysis. Multiple imputation, compared to other case deletion strategies, can provide valid inferences with less restrictive assumptions surrounding the mechanism for missing data [52-54].

As part of the secondary analyses, we plan to undertake two per-protocol analyses for the primary outcome (WOMAC composite score) at 12 months. The first analysis will include participants who did *not *have hip or knee replacement surgery. The second analysis will include participants who complied with the intervention protocol; where compliance will be defined as attendance of 80% of scheduled intervention visits, 60% of prescribed home exercise sessions in the intervention groups, and low contamination (less than 4 physiotherapy visits) in the comparison group.

#### Descriptive analyses at baseline

Descriptive statistics will be presented by intervention group at baseline to investigate the comparability between groups. This will include summary statistics of demographic, stratification, baseline measures of outcomes, and other potential confounding variables.

#### Primary analysis

Descriptive statistics for the WOMAC scale will be presented for each intervention group at 12 months follow-up. We will evaluate the effectiveness of exercise therapy and manual therapy on disability (WOMAC composite score) at 12 months using analysis of covariance (ANCOVA), a form of general linear model [55]. We will include the baseline measure as a covariate in the model, and include adjustment for the stratification variable (condition), and pre-specified potential confounding factors: age, body mass index, baseline pain intensity, duration since first diagnosis, quadriceps muscle strength, mental health, and self-efficacy [56]. These will be included regardless of whether baseline imbalance exists. This approach has been chosen because confounder selection strategies that are based on collected data can result in models with poor statistical properties [57-59]. This will adjust appropriately for any baseline imbalance and will provide the most powerful analysis [57,60]. Adjusted estimates of intervention effect from this model will be reported as the primary analysis in the trial publication.

#### Secondary analyses

Secondary analyses will be undertaken using general linear models to investigate the effectiveness of the interventions for the primary and secondary outcome variables at 9 weeks, 6 months, 12 months and 24 months. We will include the same pre-specified confounding factors as in the primary analysis, the stratification variable, and for continuous outcomes collected at both baseline and follow-up, we will include the baseline measure of the outcome. In addition, estimates of the effectiveness of exercise therapy and manual therapy from models that only include the stratification variable and baseline measure of the outcome will also be presented.

We will report the event rates for joint replacement surgery and the OMERACT-OARSI responder criteria [33,34], and the number needed to treat (NNT) statistics. Logistic regression methods and Cox's proportional hazards model will be used to compare the risk of joint replacement for each of the interventions at 12 month and 24 month follow-up. These models will include adjustment for the stratification variable.

The effect of the interventions over time will be compared using linear mixed models. These models appropriately adjust for correlation that occurs from collecting multiple observations per participant.

Finally, we will investigate (i) if there is an interaction between the two interventions for the primary outcome at 12 months, and (ii) if the effects of the two interventions differ by the condition (hip or knee osteoarthritis). For these analyses we will include appropriate interaction terms in the models [20,21]. The trial is not powered to detect these interactions and is likely to have poor precision for the interaction terms. We plan to report regression coefficients for the interaction terms and their 95% confidence intervals.

No statistical adjustment will be made for multiple testing. All tests will be two-sided and carried out at the 5% level of significance. Changes to the study design or analysis plan will be documented with full justification.

### Economic evaluation

We will assess the incremental costs and the economic consequences of delivering the manual therapy and exercise programmes compared with usual care alone according to established methods for the analysis of patient-level data [61]. We will estimate all health care consumption and costs from a societal perspective using patient self-report questionnaires, and verify hospital admissions from hospital records [62]. We will report incremental cost-utility ratios using quality-adjusted life-years derived from SF-12 scores using appropriate preference weightings [63,64].

### Trial organisation

#### Trial co-ordination and trial progress

The MOA Trial Team membership is listed in Table 2. The principal investigator will co-ordinate the trial with the assistance of a Clinical Research Administrator (CRA) and chair regular meetings of the field team and co-investigator group. The co-investigators will monitor and support the progress of the trial. The principal investigator and the field team (i.e. physiotherapists, outcome assessors, research nurse and CRA) will design the case reporting forms and MSOPs, with advice from the clinical advisors (Table 2), based on the best evidence available [5,9-14,16-19,29]. The principal investigator and CRA will manage the data flow, recording and storage.

**Table 2:** The MOA Trial team

Name	Role on trial team	Affiliation
Dr J. Haxby Abbott	Principal investigator	University of Otago
Professor G. David Baxter	Co-investigator	University of Otago
Professor A. John Campbell	Co-investigator; consultant geriatrician	University of Otago
Associate Professor M. Clare Robertson	Co-investigator; statistical analyst, economic evaluation	University of Otago
Associate Professor Jean-Claude Theis	Co-investigator; consultant orthopaedic surgeon	University of Otago
Associate Professor Peter Herbison	Statistician	University of Otago
Joanne E. McKenzie	Statistician	University of Otago; Monash University
Professor Jeffrey Basford	Research advisor	Mayo Clinic
Associate Professor G. Kelley Fitzgerald	Research and clinical advisor; advisor to PhD candidate	University of Pittsburgh
Associate Professor Timothy Flynn	Research and clinical advisor; advisor to PhD candidate	Regis University
Associate Professor Julie Fritz	Research advisor; advisor to PhD candidate	University of Utah
Dr Deidre Hurley-Osing	Research advisor	University College Dublin
Debra McNamara	Research nurse	University of Otago
Catherine Chapple	PhD candidate; outcome assessor	University of Otago
Dr Daniel Pinto	PhD candidate, economic evaluation; outcome assessor	University of Otago
Dr Alexis Wright	PhD candidate; outcome assessor	University of Otago
Martin Kidd	Physiotherapist	University of Otago
Chris Higgs	Physiotherapist	University of Otago
Jessica Smith	Physiotherapist	University of Otago
Steve Tumilty	Physiotherapist	University of Otago
Dr Ewan Kennedy	Physiotherapist	University of Otago
Dr Rhiannon Braund	Advisor to PhD candidate	University of Otago
Associate Professor Josh Cleland	Advisor to PhD candidate	Franklin Pierce College
Associate Professor Chad Cook	Advisor to PhD candidate	Duke University
Dr John Dockerty	Advisor to PhD candidate	University of Otago
Associate Professor Paul Hansen	Health economist; advisor to PhD candidate	University of Otago
Professor Helen Nicholson	Advisor to PhD candidate	University of Otago
Dr Julie Whitman	Clinical advisor	Regis University

The principal investigator will instigate and co-ordinate the training of the field team and perform audits of procedures throughout. Off-protocol and adverse event reports will be monitored monthly. Each adverse event will be independently adjudicated by two members of the MOA Trial team, blind to participant group assignment, with a third member to adjudicate in the event of disagreement. Events will be reported to the Data and Safety Monitoring Board.

#### Data and safety monitoring

Data and safety monitoring will initially be referred to the Data and Safety Monitoring Board of the Health Research Council of New Zealand. Should the Board consider the risks low and/or the recruitment period short, they may refer responsibility back to the MOA Trial Team. In this event, we will set up a panel comprising the co-investigators, selected international advisors, and an independent member to which the principal investigator will report regularly.

In the event that more than one serious adverse event of any type or class occurs that was avoidable and related to the physiotherapy programmes, we will suspend the trial. If the cause of the events cannot be determined, reconciled or remediated, we will terminate the relevant arms of the trial.

#### Data quality assurance

To enhance the accuracy of the data we will use optical mark recognition technology (Remark™, Gravic, Inc., Malvern, PA, USA) to transcribe data from paper forms to a spreadsheet, which will input data directly into a relational database (FileMaker Pro™, FileMaker, Inc., Santa Clara, CA, USA) based on a server in the Division of Health Sciences, University of Otago. These methods enable transcription error to be minimised and eliminate the need for manual double entry.

#### Intervention fidelity

Clinician adherence to the intervention protocols will be ensured by collaborative development of the protocols during a 3-day planning and training retreat, protocol-based delivery, a comprehensive MSOP, field training, structured recording forms, monthly field team meetings, audit, observation, feedback, and participant interviews.

#### Publication policy

We will submit the results of the trial for publication in an appropriate journal irrespective of outcome. We will report the trial in accordance with the CONSORT statements [65-67]. The principal investigator will be responsible for timely generation of report manuscripts, and prior to submission the co-investigators will review and approve study results papers arising from the MOA Trial. The principal investigator must approve manuscripts arising from sub-studies nested within the MOA Trial programme. Authorship of manuscripts, presentations and reports related to the MOA Trial programme will comply with the ICMJE "Uniform Requirements for Manuscripts Submitted to Biomedical Journals" [68]. Other significant contributors to the conduct of the MOA Trial will be individually named under the umbrella "the MOA Trial Team" (Table 2).

#### Timetable for the MOA Trial

June 2007 – Grant awarded by the Health Research Council of New Zealand

November 2007 – Ethical approval granted by the Lower South Regional Ethics Committee of the New Zealand Ministry of Health

10 March 2008 – Recruitment starts

March 2009 – Recruitment completed

March 2009 – First participant completes 1-year follow-up

March 2010 – Last participant completes 1-year follow-up

June 2010 – Analysis and publication of 1-year data – re: specific aims 1 & 2. Preliminary report of specific aim 3

March 2011 – Last participant completes 2-year follow-up

June 2011 – Analysis and publication of 2-year data – re: specific aim 3, longer-term report of specific aims 1 & 2.

## Discussion

The MOA Trial will be among the first randomised trials to investigate the effectiveness and cost-utility of manual therapy interventions and/or individually tailored, supervised multi-modal exercise therapy programmes for patients with hip or knee OA. It will respond to the lack of randomised trials to assess long-term outcomes or to include economic analyses. The results will help to establish the best approach to primary care of adults with mild to severe OA of the hip or knee.

## Abbreviations

ACTR: Australian New Zealand Clinical Trials Registry; ANCOVA: analysis of covariance; CRA: Clinical Research Administrator; CONSORT: Consolidated Standards of Reporting Trials; GP: General medical practitioner (family practice physician); HRC: Health Research Council of New Zealand; ICMJE: International Committee of Medical Journal Editors; ITT: intention-to-treat; MOA Trial: Management of OsteoArthritis Trial; MSOP: Manual of Standard Operating Procedures; OA: osteoarthritis; OMERACT-OARSI: Outcome Measures in Rheumatoid Arthritis Clinical Trials – Osteoarthritis Research Society International; SF-12: Short Form twelve item general health questionnaire; TENALEA: Trans European Network for Clinical Trials Services Software for Randomisation in Clinical Trials; WOMAC: Western Ontario and McMaster osteoarthritis index.

## Competing interests

The authors declare that they have no competing interests.

## Authors' contributions

JHA: conceived of the project, led the design and co-ordination of the trial, and wrote the first draft of this manuscript. JEMcK provided statistical advice and wrote the relevant sections of the manuscript. All authors participated in the trial design, commented on drafts of this paper, and read and approved the final manuscript.
